# Topographical Mapping of 436 Newly Diagnosed IDH Wildtype Glioblastoma With vs. Without MGMT Promoter Methylation

**DOI:** 10.3389/fonc.2020.00596

**Published:** 2020-05-12

**Authors:** Fatih Incekara, Sebastian R. van der Voort, Hendrikus J. Dubbink, Peggy N. Atmodimedjo, Rishi Nandoe Tewarie, Geert Lycklama, Arnaud J. P. E. Vincent, Johan M. Kros, Stefan Klein, Martin van den Bent, Marion Smits

**Affiliations:** ^1^Department of Neurosurgery, Brain Tumor Center, Erasmus MC - University Medical Center Rotterdam, Rotterdam, Netherlands; ^2^Department of Radiology and Nuclear Medicine, Erasmus MC - University Medical Center Rotterdam, Rotterdam, Netherlands; ^3^Department of Pathology, Brain Tumor Center at Erasmus MC Cancer Institute, Rotterdam, Netherlands; ^4^Department of Neurosurgery, Haaglanden MC, The Hague, Netherlands; ^5^Department of Radiology, Haaglanden MC, The Hague, Netherlands; ^6^Department of Neurology, Brain Tumor Center, Erasmus MC Cancer Institute, Rotterdam, Netherlands

**Keywords:** glioblastoma, MGMT, heatmap, atlas, localization

## Abstract

**Introduction:** O^6^**-**methylguanine-methyltransferase (MGMT) promoter methylation and isocitrate dehydrogenase (IDH) mutation status are important prognostic factors for patients with glioblastoma. There are conflicting reports about a differential topographical distribution of glioblastoma with vs. without MGMT promoter methylation, possibly caused by molecular heterogeneity in glioblastoma populations. We initiated this study to re-evaluate the topographical distribution of glioblastoma with vs. without MGMT promoter methylation in light of the updated WHO 2016 classification.

**Methods:** Preoperative T2-weighted/FLAIR and postcontrast T1-weighted MRI scans of patients aged 18 year or older with IDH wildtype glioblastoma were collected. Tumors were semi-automatically segmented, and the topographical distribution between glioblastoma with vs. without MGMT promoter methylation was visualized using frequency heatmaps. Then, voxel-wise differences were analyzed using permutation testing with Threshold Free Cluster Enhancement.

**Results:** Four hundred thirty-six IDH wildtype glioblastoma patients were included; 211 with and 225 without MGMT promoter methylation. Visual examination suggested that when compared with MGMT unmethylated glioblastoma, MGMT methylated glioblastoma were more frequently located near bifrontal and left occipital periventricular area and less frequently near the right occipital periventricular area. Statistical analyses, however, showed no significant difference in topographical distribution between MGMT methylated vs. MGMT unmethylated glioblastoma.

**Conclusions:** This study re-evaluated the topographical distribution of MGMT promoter methylation in 436 newly diagnosed IDH wildtype glioblastoma, which is the largest homogenous IDH wildtype glioblastoma population to date. There was no statistically significant difference in anatomical localization between MGMT methylated vs. unmethylated IDH wildtype glioblastoma.

## Introduction

Patients with glioblastoma have a poor prognosis with a median overall survival of 15 months, despite standard of care consisting of safe, and maximal surgical resection followed by chemo- and/or radiotherapy (1). This prognosis varies based on factors such as age, Karnofsky Performance Status, extent of resection, and molecular markers, in particular isocitrate dehydrogenase (IDH) mutation and O^6^**-**methylguanine-methyltransferase (MGMT) promoter methylation status (2).

MGMT is a DNA repair enzyme, which is expressed by the MGMT gene located on chromosome 10q26. Promoter methylation of this gene reduces MGMT protein expression and consequently decreases DNA repair and increases alkylating chemotherapy induced tumor death. Therefore, patients with MGMT methylated glioblastoma are more sensitive to neo-adjuvant temozolomide than those without MGMT methylated glioblastoma. MGMT is methylated in ~50% of patients with newly diagnosed glioblastoma (3).

There are conflicting results in the published literature on a possible differential topographical distribution of glioblastoma with vs. without MGMT promoter methylation (4). Ellingson et al. suggested that when compared with those without MGMT promoter methylation, glioblastoma with methylation are more frequently located in the left temporal lobe and less frequently in the right temporal lobe (5). However, other studies found the reverse lateralization pattern (6) or did not find any lateralization at all (7–9). These conflicting results could be ascribed to heterogeneity of molecular subtypes of glioblastoma in the studied populations, for instance when IDH wildtype glioblastoma are mixed with the genetically, and prognostically distinct IDH mutated glioblastoma, or to variation in statistical methods that were used across studies. Therefore, the question whether glioblastoma with vs. without MGMT promoter methylation have a different anatomical localization remains unanswered. In light of the updated WHO 2016 classification (10), a molecularly homogenous glioblastoma population must be used to re-evaluate the topographical distribution of MGMT methylated vs. unmethylated glioblastoma.

Therefore, we have initiated this study to re-evaluate the topographical distribution of glioblastoma with and vs. without MGMT promoter methylation in the largest homogenous IDH wildtype glioblastoma population to date.

## Methods

### Patient Inclusion

All consecutive patients aged 18 years or older newly diagnosed with a contrast-enhancing and histopathologically confirmed glioblastoma IDH wildtype who underwent tumor resection or biopsy between January 2011 and May 2018 at the Erasmus MC, University Medical Center Rotterdam, or Haaglanden MC were retrospectively included in this study. Patients were eligible if preoperative T2-weighted/fluid-attenuated inversion recovery (FLAIR) and postcontrast T1-weighted MRI scans as well as molecular data on IDH mutation and MGMT methylation status were available. Recurrent glioblastoma or confirmed IDH mutated glioblastoma were excluded. The study design was approved by the Medical Ethical Committee of Erasmus MC and Haaglanden MC. The study was performed in accordance with the 1964 Helsinki Declaration and its later amendments or comparable ethical standards.

### Image Acquisition, Tumor Segmentation, and Registration

From clinical preoperative MRI scans, which were obtained according to clinical brain tumor protocols on either a 1.5T or 3.0T scanner, T2-weighted/FLAIR and postcontrast T1-weighted images were collected. For glioblastoma segmentation, we first imported both the postcontrast T1-weighted and T2-weighted/FLAIR scans into BrainLab (BrainLab, Feldkirchen, Germany, version 2.1.0.15). We semi-automatically segmented all tumor-related contrast-enhancement (including the central necrotic part, if present) using the SmartBrush tool in Brainlab Elements and manually adapted the segmentation if needed. We then used the T2-weighted/FLAIR scan to semi-automatically segment all tumor-related non-enhancing hyperintense abnormalities (extra-lesional hemorrhage was excluded).

All tumor segmentations were then registered to the Montreal Neurological Institute (MNI) International Consortium for Brain Mapping 152 non-linear atlas. The postcontrast T1-weighted scans were registered to the T1-weighted atlas and the T2-weighted/FLAIR scans to the T2-weighted atlas. Registration was done using SimpleElastix (version 72b7e81), based on a mutual information metric using an affine registration (11). The resulting transformation parameters were used to transform the 3D segmentations to the atlas space. Registration results were visually checked to ensure that for all cases, the registered masks lay entirely within the brain mask of the atlas. No adjustments were made to the initial registration settings for individual patients. We created voxel-wise frequency maps for all glioblastoma combined, and frequency difference maps of glioblastoma with vs. without MGMT promoter methylation.

### Molecular Analysis

Tumor tissue samples were obtained from patients through surgical resection or biopsy. Histopathological examination was performed by neuropathologists. DNA was extracted from microdissected FFPE tissue fragments by proteinase K digestion for 16 h at 56°C in the presence of 5% Chelex 100 resin and used after inactivation of proteinase K and removal of cell debris and the Chelex resin. IDH mutational analysis was assessed with Sanger sequencing and targeted next generation sequencing (NGS) analysis. Sanger sequencing of PCR-amplified fragments from IDH1 and IDH2 mutational hot spots was essentially performed as previously described (12). M13-tailed primers for PCR amplification of IDH1 codon 132 were forward 5′- TCTTCAGAGAAGCCATTAT-3′ and reverse 5′-GCAAAATCACATTATTGCCAAC-3′, for IDH2 codon 140, forward 5′-GGCTGCAGTGGGACCACTAT-3′ and reverse 5′- TTGGTCCAGCCAGGGACTAG-3′, and for IDH2 codon 172, forward 5′- ACATCCTGGGGGGGACTGTC-3′ and reverse 5′- GACAAGAGGATGGCTAGGCG-3′. The M13-tail for the forward primers was: 5′- TGTAAAACGACGGCCAGT-3′ and for the reverse primers: 5′-CAGGAAACAGCTATGACC-3′. After initial denaturation at 95°C for 3 min, 35 cycles of 95°C for 15 s, 60°C for 15 s, and 72°C for 15 s were performed, followed by 7 min at 72°C. Subsequent sequence analyses of the PCR products was carried out with M13 forward and reverse primers on a 3730 XL Genetic Analyzer (Applied Biosystems, Foster City, CA, USA).

Targeted NGS was performed by semiconductor sequencing with the Ion Torrent platform using supplier's materials and protocols (Thermo Fisher Scientific) with a dedicated panel for detection of glioma-specific aberrations, including IDH1 and IDH2 hot spot mutations essentially as previously described (13). Library and template preparations were performed consecutively with the AmpliSeq Library Kit 2.0-384 LV and the Ion PGM Template OT2 200 kit. Sequencing was performed with the Ion PGM Sequencing 200 Kit v2 on an 318v2 chip with the PGM system. Data were analyzed with the Torrent variant caller (Thermo Fisher Scientific), and variants were annotated in a local Galaxy pipeline using ANNOVAR. Data were collected during several years using different glioma panels. Sequenced areas of IDH1 codon 132 and IDH2 codons 140 and 172 are given in the supplementary data of Dubbink et al. (13).

MGMT promoter methylation status was assessed by methylation-specific PCR essentially as described by Esteller et al. (14) Bisulfite conversion and subsequent purification are performed with the EZ DNA Methylation-Lightning Kit (Zymo Research) according to the supplier's protocol. Methylation-specific PCR was performed with primers specific for either methylated or the modified unmethylated DNA. Converted primer sequences for unmethylated DNA were forward 5′-TTTGTGTTTTGATGTTTGTAGGTTTTTGT-3′ and reverse 5′-AACTCCACACTCTTCCAAAAACAAAACA-3′, and for the methylated reaction, forward 5′-TTTCGACGTTCGTAGGTTTTCGC-3′ and reverse 5′-GCACTCTTCCGAAAACGAAACG-3′. PCR was performed after initial denaturation at 96°C for 5 min, 40 cycles of 92°C for 45 s, 59°C for 65 s, and 72°C for 45 s, followed by 7 min at 72°C. Five microliters of each 15 μl methylation-specific PCR product was loaded onto a 1.5% agarose gel stained with GelRed (Biotium) and examined under ultraviolet illumination. SW48 cell line DNA and tonsil DNA was used as a positive control for methylated and unmethylated alleles of MGMT, respectively. Controls without DNA were used for each set of methylation-specific PCR assays.

### Statistical Analysis

We first tested the differences between preoperative enhancing and non-enhancing tumor volumes as well as their ratio with the Kruskal-Wallis test. We mapped the anatomical localization of all MGMT methylated and unmethylated glioblastoma by iterating over all voxels in the MNI atlas and counting the number of tumor frequencies for each group in each voxel. To test for differences in spatial distribution between glioblastoma with vs. without MGMT promoter methylation, we assessed the cluster-wise significance at the voxel-level between distributions, using permutation testing with Threshold Free Cluster Enhancement (15) in the software package “FSL Randomise” (version 5.0.9, using 10,000 permutations) (16). We also performed the same analysis with correction for age as a potential confounder by determining age for each patient at the time of the MRI scan. We then calculated the difference between each patient's age and the average age of all patients included in the study, which was added to the experimental setup for FSL Randomise. Threshold Free Cluster Enhancement corrects *p*-values for the family-wise error in testing multiple voxels, considering a corrected *p* < 0.05 as statistically significant.

## Results

In total, 769 patients with newly diagnosed, contrast enhancing glioblastoma were screened, of whom we excluded 333 patients: 22 were excluded due to IDH mutation and 311 were excluded due to insufficient or missing molecular data on IDH mutation or MGMT methylation status. Final analysis included 436 patients with IDH wildtype glioblastoma (see flowchart, Supplementary Material); 211 with and 225 without MGMT promoter methylation. Three hundred forty patients had undergone a surgical tumor resection and 96 a diagnostic biopsy. In all patients, preoperative postcontrast T1-weighted MRI scans were available; in 90 patients, T2-weighted FLAIR scans, and in 346 patients T2-weighted scans were available. When compared with MGMT unmethylated glioblastoma, MGMT methylated glioblastoma had a significantly higher ratio of non-enhancing vs. contrast-enhancing volume [2.09 (inter quartile range 2.6) and 2.5 (inter quartile range 3.3), *p* = 0.045, respectively]. Patient and tumor characteristics are further presented in Table 1.

**Table 1:** Patient and tumor characteristics.

**Characteristics**	***n***	**%**	
All patients	436	100	
**Sex**			
Male	276	63.3	
Female	160	36.7	
**Age**			
≤ 65	227	52.1	
>65	209	47.9	
Mean, years (SD)	61.5 (16.2)		
**Karnofsky performance status**			
≤ 70	142	32.6	
>70	294	67.4	
Mean (SD)	80 (12.5)		
**Preoperative MRI scans**			
T1 postcontrast	436	100	
T2-weighted	346	79.4	
T2-weighted FLAIR	90	20.6	
**Neurosurgical procedure**			
Resection	340	78.0	
Biopsy	96	22.0	
**Preoperative volume, median cm**^**3**^ **(IQR)**	**MGMT promoter**		***p*****-value**
	**Methylated 211 (48.4%)**	**Unmethylated 225(51.6%)**	
Contrast-enhancing	30.1 (39.5)	35 (45.8)	0.130
Non-enhancing	75.5 (105.0)	65.5 (84.2)	0.338
Non-enhancing/contrast-enhancing ratio	2.5 (3.3)	2.09 (2.6)	0.045

*SD, standard deviation; IQR, interquartile range; CE, contrast enhancement; FLAIR fluid-attenuated inversion recovery*.

### Topographical Mapping of 436 IDH Wildtype Glioblastoma

**Figure 1:**
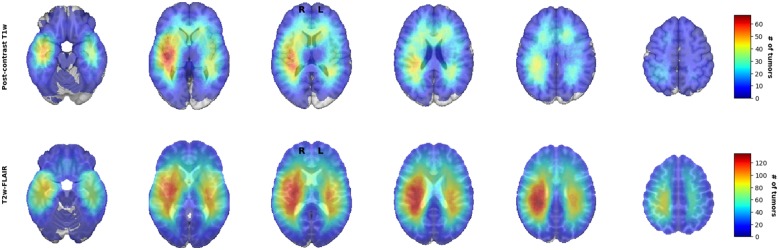
Heatmaps of all 436 isocitrate dehydrogenase (IDH) wildtype glioblastoma.

**Figure 2:**
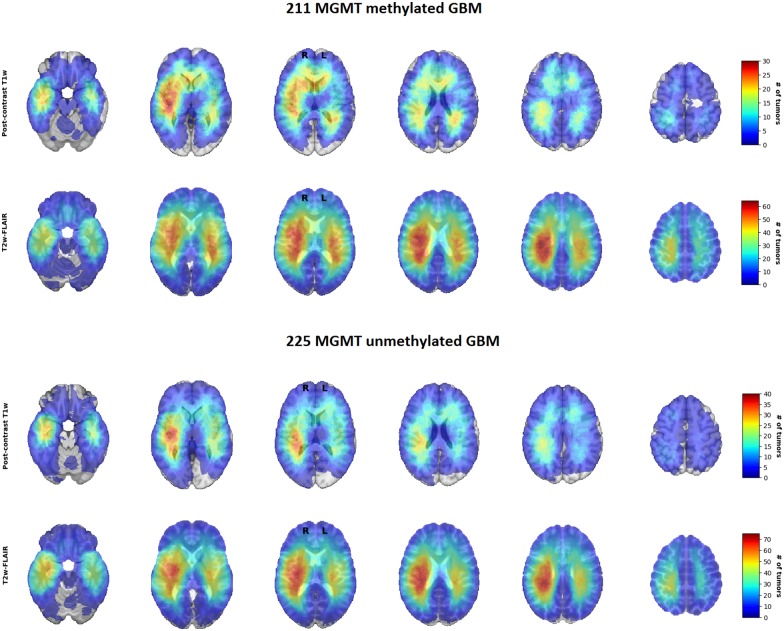
Heatmaps of O^6^-methylguanine-methyltransferase (MGMT) methylated (*N* = 211) and unmethylated (*N* = 225) glioblastoma.

**Figure 3:**
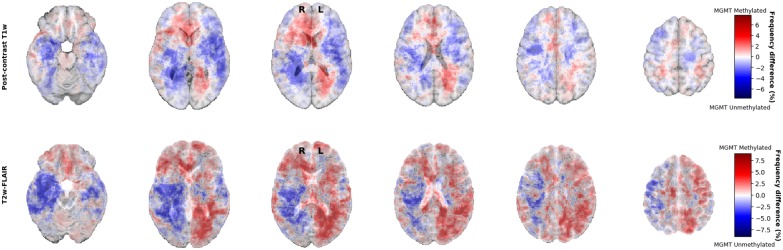
Frequency difference maps between MGMT methylated (*N* = 211) and unmethylated (*N* = 225) glioblastoma.

For visual inspection, heatmaps based on postcontrast T1-weighted and T2-weighted/FLAIR segmentations were created for all 436 patients combined (Figures 1, 2), as well as frequency difference maps between MGMT methylated vs. unmethylated glioblastoma (Figure 3). Visual inspection of maps in Figure 1 suggests that glioblastoma were most frequently located in the right temporal, insular, and parietal area, and near the periventricular area both frontally and occipitally. Visual inspection of Figures 2, 3 indicates that when compared with MGMT unmethylated glioblastoma, methylated glioblastoma were more frequently located near bifrontal and left occipital periventricular area (up to 6.5% frequency difference) and less frequently near the right occipital periventricular area (up to 9.1% frequency difference).

**Figure 4:**
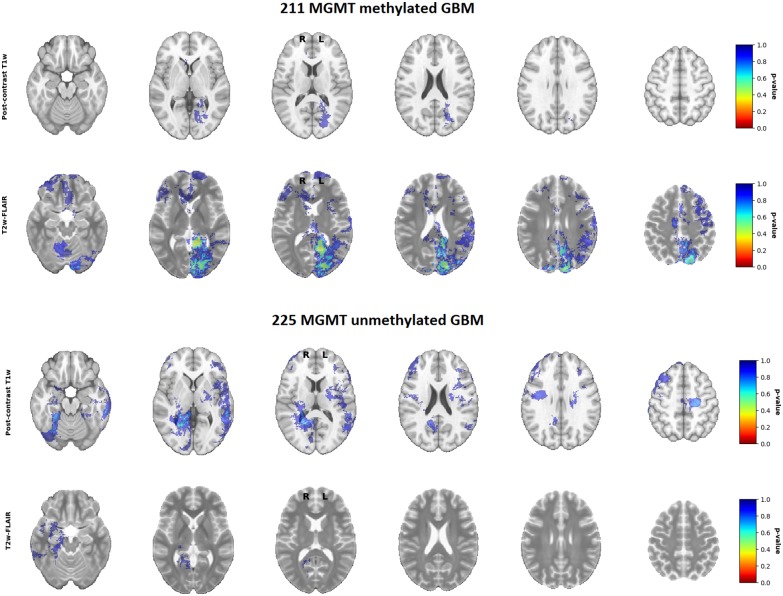
*P*-value maps of MGMT methylated (*N* = 211) and unmethylated (*N* = 225) glioblastoma.

To test whether this difference was statistically significant, voxel-wise analyses of both the postcontrast T1-weighted and T2-weighted/FLAIR segmentation heatmaps were performed. Although statistical analysis of the postcontrast T1-weighted scans marked a region near the right occipital periventricular area as a potentially discriminating area between MGMT methylated vs. unmethylated glioblastoma, this difference was not statistically significant (Figure 4, together with corresponding *p*-values). This figure in fact shows that not any statistically significantly discriminating brain area between MGMT methylated and unmethylated glioblastoma could be found (*p* < 0.05). This result did not change after an additional analysis with correction for age as potential confounding factor. Scroll-through video clips for visual inspection of all topographic maps are publicly available as Supplementary Material.

## Discussion

This study voxel-wise analyzed postcontrast T1-weighted and T2-weighted/FLAIR heatmaps and showed that there was no statistically significant difference in anatomical localization between MGMT methylated vs. unmethylated IDH wildtype glioblastoma.

The primary reason to initiate this study was to re-evaluate the anatomic localization of MGMT methylated vs. unmethylated glioblastoma in light of the updated WHO 2016 classification era following conflicting reports on this topic (4). Ellingson et al. (17) reported that glioblastoma with MGMT methylation were lateralized to the left hemisphere (temporal lobe) and that those without were lateralized to the right hemisphere (17), which was in line with their previous article (2012) and in which they included a substantial portion of their previously studied glioblastoma population (5). However, in contrast to these findings, there are also studies that found the reverse pattern of hemispheric lateralization, in which glioblastoma with MGMT methylation were located more frequently in the right hemisphere, while those without MGMT methylation lateralized to the left hemisphere (6). Additionally, there are conflicting reports on lobar distribution, in which glioblastoma with MGMT methylation were more frequently located in the parietal and occipital lobes, while those without were located more frequently in the temporal lobes (8). A recent study suggested after qualitative analyses that subventricular zones were more frequently spared with MGMT methylated glioblastoma, but found no difference in hemispheric lateralization between glioblastoma with and without MGMT promoter methylation (9). Finally, there are also studies that report no differences in localization between glioblastoma with and without MGMT methylation (7, 18) in concordance with the findings of our study.

These conflicting results in the literature can potentially be ascribed to two methodological issues. First, inconsistencies may arise from variations in glioblastoma patient populations across studies, many of which were performed in the pre-WHO 2016 classification era when the impact of molecular subtyping of glioblastoma according to IDH mutation status was less of a consideration (10). Ellingson et al. (17) included a series of 507 *de novo* glioblastoma with mixed IDH subtypes, including 366 IDH wildtype, 34 IDH mutated glioblastoma, and also 107 glioblastoma without data on IDH mutation status (17). Moreover, the majority of the studies did not report the IDH mutation status of included glioblastoma (5, 6, 8, 18).

Mixing molecular subtypes or not knowing IDH mutation status of glioblastoma is undesirable when assessing topographical distribution of molecular subtypes (10), since it is now known that IDH mutated glioblastoma represent a distinct molecular subtype of glioblastoma from a distinct precursor lesion which have a predominantly frontal lobe involvement when compared with IDH wildtype glioblastoma (19). This topographic link between IDH mutation and MGMT methylation was also suggested by Ellingson et al. (17) by demonstrating that IDH mutated and MGMT methylated glioblastoma were indeed more frequently localized in the frontal lobe (17). This has not only been demonstrated in glioblastoma but also in non-contrast enhancing low grade glioma in which IDH mutated low grade glioma (both oligodendroglioma and astrocytoma) were more frequently located in the frontal lobes, while non-contrast enhancing IDH wildtype astrocytoma were more frequently located in the basal ganglia of the right hemisphere (20). This topographical link thus suggests IDH mutation status as a (confounding) factor between MGMT methylation status and localization. Therefore, studies must be conducted based on homogeneous tumor populations with respect to IDH mutational status. This hypothesis was recently supported by Roux et al., who assessed a homogenous IDH wildtype glioblastoma population (*n* = 392) and found no difference in localization between glioblastoma with and without MGMT methylation, in line with our study (21).

Second, the conflicting results in the literature may arise from different statistical methods that were used across studies. Studies often investigated the anatomic localization of glioblastoma with and without MGMT promoter methylation with visual examination, qualitatively, without a statistical, voxel-wise quantitative analysis (7–9, 18). Ellingson et al. (17) used frequency difference maps to demonstrate that MGMT methylated glioblastoma were more frequently localized in the left temporal lobe (17). Using similar frequency difference maps, we also found topographical differences, which indicated that when compared with MGMT unmethylated glioblastoma, MGMT methylated glioblastoma were more frequently localized near bifrontal and right occipital periventricular area and less frequently near the right occipital periventricular area. However, we showed that these apparent differences did not survive rigorous statistical testing. Ellingson et al. report the use of “Analysis of Differential Involvement” for their statistical analysis, which is based on the Fisher exact test (5). We used “FSL randomise,” which is different from the Fisher exact test because it does not make any assumptions about the underlying distribution of the variables (16). Another methodological difference can be found in the correction for multiple comparisons. Ellingson et al. used random permutations based on Bullmore et al. instead of the more recently proposed and widely accepted method of doing random permutations employed in “FSL randomise” based on Smith and Nichols (15) and Bullmore et al. (22). Furthermore, the method by Bullmore et al. requires a user-defined threshold for clustering, which can impact the results substantially (22). Instead, we used “Threshold Free Cluster Enhancement,” which does not require thresholding to determine the clusters, and which has been shown to have a higher sensitivity compared to other methods (15). Our stringent methodology of rigorous statistical testing and applying new insights in glioblastoma molecular subtyping to a large studied patient population are the strengths of our study.

## Limitations

The main limitation of this study is its retrospective design, which may have introduced selection and confounding biases. Selection bias may occur when patients who receive diagnostic biopsies are excluded from analysis, since these tumors are often large, multifocal, located deep within the basal ganglia, or crossing midline. This may skew the results on tumor localization of glioblastoma, which is our main outcome. We have therefore attempted to limit this bias first by consecutive inclusion of all glioblastoma patients operated upon between 2011 and 2018 in our cohort, including diagnostic biopsies. In addition, it is known that tumor localization is associated with IDH mutation status, with IDH mutated tumors located more frequently in the frontal lobes, as mentioned earlier (19). Since IDH mutation status is both associated with tumor localization and MGMT methylation status, it may function as a confounding factor. We therefore have also attempted to limit this potential bias by excluding all IDH mutated tumors. Another limitation is that we included patients from two medical centers from a period of over seven years. This introduced variation of MRI scan protocols such as magnet strength, voxel size, and slice thickness, which consequently may have negatively influenced registration accuracy and anatomical localization. Such registration inaccuracies can however be considered minor relative to the size of the tumor, and it is therefore unlikely that our results were significantly impacted by scanner variations. Additionally, tumor volume assessment on these MRI scans were performed by one observer without confirmation of a second, independent assessor. This may have introduced some degree of information bias. We have attempted to limit this bias during volumetric assessment by blinding the assessor for patients' clinical and molecular characteristics. Also, it is known that both the inter- and intraobserver agreement for preoperative tumor volumes in glioblastoma is relatively high, while small variations in segmentation will probably have only a very limited effect on determining gross tumor localization (23). Finally, it should be noted that the known intertest variability is a limitation of MGMT analyses, as assays used in other studies may produce slightly different MGMT methylation results (24). This may partially explain the variety in the proportion of MGMT methylated tumors reported in literature.

To conclude, in the largest homogenous IDH wildtype glioblastoma population to date, we showed that visual appearance of differences could not be confirmed with rigorous voxel-wise statistical testing and thus that there is no statistical difference in anatomical localization between IDH wildtype glioblastoma with vs. without glioblastoma promoter methylation.

## Data Availability Statement

The raw data supporting the conclusions of this article will be made available by the authors, without undue reservation, to any qualified researcher.

## Ethics Statement

The studies involving human participants were reviewed and approved by MEC-2019-0641. Written informed consent for participation was not required for this study in accordance with the national legislation and the institutional requirements.

## Author Contributions

FI, SV, SK, MB, and MS: literature search, study design, data collection, data analysis, data interpretation, and writing. HD, PA, RN, GL, AV, and JK: data collection, data interpretation, and writing.

## Conflict of Interest

The authors declare that the research was conducted in the absence of any commercial or financial relationships that could be construed as a potential conflict of interest.

## Supplementary Material

Supplementary Figure S1 Flowchart.

Supplementary Video S1 Frequency difference maps T1w.

Supplementary Video S2 Frequency difference maps T2w.

Supplementary Video S3 Heatmap all GBM T1w.

Supplementary Video S4 Heatmap all GBM T2w.

Supplementary Video S5 Heatmap methylated GBM T1w.

Supplementary Video S6 Heatmap methylated GBM T2w.

Supplementary Video S7 Heatmap unmethylated GBM T1w.

Supplementary Video S8 Heatmap unmethylated GBM T2w.

Supplementary Video S9 *P* value map methylated GBM T1w.

Supplementary Video S10 *P* value map methylated GBM T2w.

Supplementary Video S11 *P* value map unmethylated GBM T1w.

Supplementary Video S12 *P* value map unmethylated GBM T2w.

## Abbreviations

FLAIR: fluid-attenuated inversion recovery
IDH: isocitrate dehydrogenase
MGMT: O^6^-methylguanine-methyltransferase.
