# Supplementary figures and images for: Topographical Mapping of 436 Newly Diagnosed IDH Wildtype Glioblastoma With vs. Without MGMT Promoter Methylation
Source: Front Oncol. 2020 May 12;10:596. doi: 10.3389/fonc.2020.00596 (PMC7235346; doi:10.3389/fonc.2020.00596)

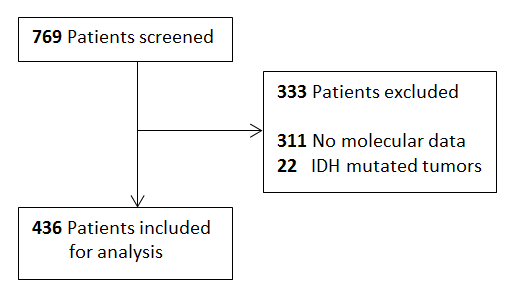

Supplement: Supplementary Figure S1 — Flowchart. [file Image_1.TIF]
